# Prevalence and Predictors of Depression, Anxiety, and Stress among Youth at the Time of COVID-19: An Online Cross-Sectional Multicountry Study

**DOI:** 10.1155/2020/8887727

**Published:** 2020-10-06

**Authors:** Omar Al Omari, Sulaiman Al Sabei, Omar Al Rawajfah, Loai Abu Sharour, Khalid Aljohani, Khaled Alomari, Lina Shkman, Khloud Al Dameery, Ahmed Saifan, Bushara Al Zubidi, Samh Anwar, Fadwa Alhalaiqa

**Affiliations:** ^1^Sultan Qaboos University, College of Nursing, Muscat, Oman; ^2^Curtin University, School of Nursing, Midwifery and Paramedicine, Perth, Western Australia, Australia; ^3^College of Nursing, Al al-Bayt University, Jordan; ^4^ALZaytoonah University of Jordan, College of Nursing, Amman, Jordan; ^5^Taibah University, College of Nursing, Saudi Arabia; ^6^Applied Science Private University, Amman, Jordan; ^7^Baghdad University, College of Nursing, Baghdad, Iraq; ^8^Alexandria University, College of Nursing, Alexandria, Egypt; ^9^Philadelphia University, College of Nursing, Amman, Jordan

## Abstract

Depression and anxiety are prevalent mental illnesses among young people. Crisis like the Coronavirus Disease 2019 (COVID-19) pandemic may increase the current prevalence of these illnesses. A cross-sectional, descriptive design was used to (1) explore the prevalence of depression, anxiety, and stress among youth and (2) identify to what extent certain variables related to COVID-19 could predict depression, anxiety, and stress (DAS) among young people in six different countries. Participants were requested to complete an online survey including demographics and the DAS scale. A total of 1,057 participants from Oman (*n* = 155), Saudi Arabia (*n* = 121), Jordan (*n* = 332), Iraq (*n* = 117), United Arab Emirates (*n* = 147), and Egypt (*n* = 182) completed the study. The total prevalence of depression, anxiety, and stress was 57%, 40.5%, and 38.1%, respectively, with no significant differences between countries. Significant predictors of stress, anxiety, and depression were being female, being in contact with a friend and/or a family member with mental illness, being quarantined for 14 days, and using the internet. In conclusion, COVID-19 is an epidemiological crisis that is casting a shadow on youths' DAS. The restrictions and prolonged lockdowns imposed by COVID-19 are negatively impacting their level of DAS. Healthcare organisations, in collaboration with various sectors, are recommended to apply psychological first aid and design appropriate educational programmes to improve the mental health of youth.

## 1. Introduction

Since the beginning of 2020, Coronavirus Disease 2019 (COVID-19) has headed global news. The story started back in December 2019 in Wuhan, China, when an outbreak of cases infected with a novel, deadly virus was reported. Later, the causing microorganism was found to be a new type of coronavirus, and the disease was labelled COVID-19 [1]. Between January and March 2020, the disease spread to more than 110 countries and the number of cases outside China increased 13-fold [2]. As a result, the World Health Organisation (WHO) declared COVID-19 a pandemic on 11 March 2020 [3]. As of 5 May, there were over 3.6 million confirmed cases and over 251,898 deaths worldwide, according to the Johns Hopkins Coronavirus Resource Center [4]. Countries initiated a series of measures to break the chain of infection and control the pandemic, including local and international travel bans, bans on large gatherings, suspension of public transport, closure of schools and universities and of business, social distancing, stay-at-home orders, and curfews [5]. These restrictions and the uncertain trend of the disease can significantly affect mental wellbeing. The educational [6] and economic [7] sectors were almost paralysed, and healthcare systems were overwhelmed by the flood of new cases. The pandemic has become the central concern of people around the world, and they spend most of their time watching and reading the news around it. In general, people are afraid, angry, anxious, and stressed [8].

Youth is defined as age 15 to 24 years, and it includes middle and late adolescence [9]. It is characterised by ongoing changes in physical, psychological, and social dimensions [10]. For healthy growth and development, youth needs to have a sense of belonging, love, achievement, and independence and to have a purpose in life [10]. During this developmental stage, many types of behaviour are developed which can lead to either normalcy or mental health illness [11, 12]. Depression, anxiety, and stress (DAS) are the most common mental illnesses among youth [13]. There are shared symptoms between anxiety and depression. However, psychologists can differentiate DAS diagnoses using a tripartite model [14]. This model postulates that anxiety and depression share common characteristics of negative effect but can be distinguished by low positive effect associated with depression and high physiological hyperarousal associated with anxiety [14]. In previous studies, the prevalence of stress among Iraqi youths was found to be 51.1% [15]; the prevalence of anxiety among youths in Oman, Jordan, Egypt, Iraq, and Saudi Arabia was 17% [16], 42.1% [17], 41.2% [15], and 63.5% [18], respectively; and the prevalence of youth depression was 73.8% in Jordan [17], 28.6% in Egypt [19], 17% in Oman [16], 29.4% in Iraq [15], and 71% in Saudi Arabia [18]. Given such alarming statistics, the American Academy of Child and Adolescent Psychiatry (AACAP) recommends regular screening of young people for mental illness [20].

Youth in the Middle East have common backgrounds as the majority speak Arabic and are Muslims. Moreover, COVID-19 has impacted all Middle Eastern countries, which have taken similar measures to restrain the pandemic including suspension of schools and universities and stay-at-home orders. Giving mental illness special attention at this time is crucial. Several studies have linked crises to developing mental illnesses such as DAS [21, 22]. If left undiagnosed and untreated, mental illness can negatively impact the development, social life, and even future careers of young people [23]. Positive relationships have been found between DAS and poor academic achievement [24], poor peer friendship [25], substance misuse [26, 27], and suicide attempts [28]. The impact of COVID-19 may play a significant role in triggering or worsening signs, symptoms, and eventual development of mental illness. With limited knowledge about COVID-19, the uncertainty of its trends, the worry over getting the disease itself, and drastic changes in lifestyles and livelihoods, the mental health of youth has become a serious concern [29]. The purpose of this study was to explore the prevalence of DAS among youth from six Middle Eastern countries and to identify the extent to which some COVID-19-related variables could predict DAS among them.

## 2. Materials and Methods

A cross-sectional descriptive design was used to assess the relationship between DAS during the COVID-19 pandemic. The target population was young people aged 15 to 24, as defined by the United Nations [9]. Between 1 and 15 April 2020, an online survey was distributed using social media platforms to recruit participants in 11 countries: Oman, Kuwait, Saudi Arabia, UAE, Qatar, Bahrain, Iraq, Jordan, Lebanon, Palestine, and Egypt. However, respondents from only six countries completed the survey. WhatsApp and Facebook were the main social media platforms used in the study settings. The inclusion criteria were (1) willingness to participate, (2) being 15 to 24 years old at the time of the study, (3) ability to read and type in Arabic, and (4) residence in one of the countries included in the study at the time of the survey.

### 2.1. Sample Size

To have a medium effect size (Cohen's f2 = 0.15), statistical power is 80%, probability level is 0.05, and the estimated sample size from each country is 113 study participants [30]. 113 participants from each country were required to take part in the study.

### 2.2. Ethical Approval

Ethical approval was obtained from the study sites prior to data collection, and consent was assumed as completing the survey questions. Participants were informed that their participation was voluntary and that they could withdraw from the study at any point or choose not to answer any question. Participants' confidentiality was maintained as no identifying information was collected and findings will be disseminated only in aggregate.

### 2.3. Study Variables and Measurements

A structured questionnaire was used to collect information about (1) participants' sociodemographics including age; gender; educational level and type; presence of family members, friends, or colleagues with COVID-19; previous history of depression or anxiety; history of medication for depressive syndrome; frequency of watching news about COVID-19; and internet use; (2) depression; (3) anxiety; and (4) stress.

Several scales are available to measure depression symptoms, including the depression scale [31, 32] and Beck depression inventory-II [33]; however, the researchers made a deliberate decision to use the Depression, Anxiety, and Stress Scale (DASS) because it is available in the public domain, the Arabic version has been validated, it is sensitive to youth, and it measures depression, anxiety, and stress in the same survey. The short form of DASS is a 21-item, 4-point Likert scale where 0 = does not apply to me at all; 1 = applies to me to some degree, or some of the time; 2 = applies to me to a considerable degree, or a good part of my life; and 3 = applies to me very much, or most of the time.

The depression scale assesses a range of depressive syndromes including dysphoria, hopelessness, and lack of interest/involvement. The higher score indicates a higher level of depression, categorised by scores as normal (0-9 points), mild [10–13], moderate [14–20], and severe [21–27]; scores of 28 and above indicate extremely severe depression [34].

The anxiety scale assesses the subjective experience of the anxiety effect, autonomic arousal, skeletal muscle effects, and situational anxiety. Scores are classified as normal (0-7 points), mild [8, 9], moderate [10–14], and severe [15–19], with scores of 20 or more indicating extremely severe anxiety [34].

The stress scale assesses difficulty relaxing, being irritable/overreactive and impatient, and being easily upset/agitated: normal (0-14), mild [15–18], moderate [19–25], and severe [26–33], with scores of 34 or more indicating extremely severe stress [34].

The Arabic version of DASS is available in the public domain [34]. The reliability (Cronbach′s alpha = 0.88) and construct validity of the Arabic version are well established [35].

### 2.4. Statistical Analysis Plan

Data were transferred from Google Forms to an Excel sheet and exported to SPSS version 23 for the analysis. Descriptive statistics were used to determine the frequencies, mean, and standard deviation and to describe sample demographics. Multivariate linear regression was conducted to identify the extent to which certain variables related to COVID-19 predict DAS among the participants. Statistical significance was set a priori at *p* < 0.05.

## 3. Results

Over two weeks of data collection, 1,057 participants from six countries completed the survey. The majority of participants were female 756 (71.5%), and the mean age was 21.01 (SD = 1.69). Most (1,048, 99.1%) had never been diagnosed with mental illness and 91 (8.6%) with chronic illnesses. The majority of participants were university students (762, 72.1%), with only 29 (2.7%) in 9th grade. On average, participants had spent 9.74 hours (SD = 4.99) daily surfing the internet since COVID-19 was declared as a pandemic, compared with only 5.64 hours (SD = 3.83) daily before the outbreak. Almost two-thirds of the participants were following COVID-19 news by surfing the internet (685, 64.8%) (see Table 1).

One-way ANOVA revealed that depression, anxiety, and stress were not statistically different between the six countries (*F* = 0.996, *p* = 0.419; *F* = 1.979, *p* = 0.079; and *F* = 1.841, *p* = 0.102, respectively). The prevalence of depression, anxiety, and stress was 57%, 40.5%, and 38.1% among all participants. Participants from Saudi Arabia had the lowest rates, and Egyptian participants the highest prevalence (Table 2).

### 3.1. Multivariable Analysis

Three models were built to identify the predictors of depression, anxiety, and stress. The normality, linearity, heteroscedasticity, and independence of residuals were assessed. Bivariate analysis using an independent *t*-test was performed to assess whether there was a significant difference in the means between the independent variables and the three dependent variables depression, anxiety, and stress, with other explanatory factors (Table 3). The Spearman correlation coefficient was used to test for bivariate correlation between the number of hours spent on the internet and the three dependent variables. The results revealed that depression, anxiety, and stress were positively associated with the number of hours spent on the internet after COVID-19 with *r* coefficient of 0.15, 0.13, and 0.17 (*p* > 0.01), respectively. Only variables with statistically significant associations with the dependent variables were entered into the preliminary regression model (For more detail, see Table 3). The backward approach was employed, and explanatory variables which were not significant were excluded to build the final linear regression model.

#### 3.1.1. Depression

The final model was statistically significant compared to the constant (*F* = 18.199, *p* < 0.001) with final variables of female gender, having a family member with mental illness, having a friend with mental illness, exposed to an infected person with COVID-19, being quarantined for 14 days, using the internet after COVID-19, and being at risk of infection as significant predictors of depression. *R*^2^ and adjusted *R*^2^ of the final model were 0.108 and =0.102, respectively (Table 4, model 1).

#### 3.1.2. Anxiety

The final model was statistically significant compared to the constant (*F* = 23.4, *p* < 0.001). Significant predictors of anxiety included female gender, having a friend with mental illness, exposed to a person with COVID-19, using the internet after COVID-19, and being at risk of infection. *R*^2^ and adjusted *R*^2^ of the final model were 0.100 and =0.096, respectively (Table 4, model 2).

#### 3.1.3. Stress

The final model was statistically significant compared to the constant (*F* = 23.4, *p* < 0.001). Significant predictors of anxiety included female gender, having a family member with mental illness, having a friend with mental illness, having a relative diagnosed with COVID-19, using the internet after COVID-19, and being at risk of infection with COVID-19. *R*^2^ and adjusted *R*^2^ of the final model were 0.122 and 0.117, respectively (Table 4, model 3).

## 4. Discussion

The aim of the current study was to explore the prevalence of DAS among youth in six Middle Eastern countries and to identify the associated predictors. The prevalence of anxiety was found to range from 33.1% in Saudi Arabia to 51.6% in Egypt, with a total prevalence of 40.5% in the six countries. These results are consistent with previously reported prevalence rates [16, 17, 19]. The prevalence of depression in the current study ranged from 47.9% in Saudi Arabia to 64.8% in Egypt with a total prevalence of 57%. In previous studies, the reported rates of depression in Egypt [19], Oman [16], and Iraq [15] were lower than those identified in this study. However, the depression rates in Jordan [17] and Saudi Arabia [18] were higher than the identified rates in this study. The inconsistency of some prevalence rates of DAS with previous literature may be related to different survey methods, recruitment of young people with different age groups, and use of different tools. The findings of the current study were similar to those of previous studies, which suggests that the COVID-19 pandemic has no significant impact on the selected DAS variables.

One possible explanation is that at the time of this study, the epidemic had not peaked, and the number of reported cases was relatively insignificant in the study settings. In order for people to be psychologically affected, two of the following four conditions should be met: (1) a large number of injuries and casualties should be reported, (2) mass damage to property, (3) disruption of social support, and [4] ongoing economic problems [36]. At the time of the data collection, most of these conditions were not present, which may explain the relatively similar prevalence rates of DAS among youth before and after COVID-19.

In the current study, several factors were associated with DAS prevalence rates. Some of these variables were also reported in previous literature, including female gender, having a friend and/or family member diagnosed with mental illness, and using the internet. Young females have higher levels of DAS [37, 38]. Mental illnesses affect not only the diagnosed person but also the surrounding people, such as family members and friends. Research studies have found that children who are in close contact with mentally ill people are at a greater risk of developing mental illness themselves [39, 40]. There is a need to establish a support system for young people living in families with mentally ill members. The use of the internet was found to be a predictor of DAS in this study. The lockdown, suspension of studies, and moving to online learning have significantly increased the use of the internet among youth from an average of 5.46 hours a day before COVID-19 to 9.74 hours a day. The current finding is well reported in the literature [41]. Therefore, healthcare organisations in collaboration with ministries of telecommunications are strongly encouraged to design and provide specific psychological promotion programmes for youth during this pandemic with the aim of promoting their mental health.

Income, age, academic status, study in a capital city, and health-related condition were other predictors identified in previous literature [42–44] but not measured in the current study. Older students are at greater risk of DAS [43]. Youth with health and academic problems were found to have greater stress [42]. Those living in low- and middle-income families have higher rates of DAS than those in high-income families [43]. In the current study, the majority of participants declined to answer the income question, and therefore, it was not included in the analysis. However, it was interesting to observe that the rate for DAS among youth from high-income countries such as Oman, Saudi Arabia, and UAE was comparable to that from lower-income countries such as Jordan, Egypt, and Iraq. It is clear that DAS is a mixture of biological, personal, spiritual, emotional, and social factors and that income is only one factor. There is a need to regularly screen youth and identify the newly evolved variables associated with DAS [20].

New variables identified in the current study that are directly related to the current epidemiological crisis have not been reported previously. These include contacting a person with COVID-19, being quarantined for 14 days, being at risk of infection from COVID-19, and the increased use of the internet after COVID-19's appearance. The ambiguity of COVID-19, being labelled as a deadly disease, the disruption to daily activity, and believing being at risk from COVID-19 were identified as factors that increase the level of fear and DAS among youth. This finding is supported by previous research studies [45, 46]. Another study found that the stress level of young people who had been quarantined was four times higher than that of their counterparts who had not been [47]. Families can play a significant role in minimising the effect of staying at home. Parents need to let young people express their feelings and fears about the current situation. They also need to make an effort to increase family time to provide youths with a sense of security.

With stay-at-home orders and restrictions, governments should also consider finding new technological methods to prevent, screen, and manage potential mental illness. Online mental health services should be activated, as has been done in some countries [48]. Governments should adopt the same approach and establish online mental health clinics where people can be virtually screened and consulted. These clinics can help individuals to stay anonymous and encourage them to convey their feelings in a free environment. Another role for these clinics is to develop psychological programmes and educate young people about mental illnesses and how to learn about and counter them [49] and about COVID-19. Providing mental healthcare virtually can help in reducing the prevalence of DAS. Countries need to establish creative methods to shift the attention of youth. For example, the Jordanian government created an online competition for the general public in order to distract them, and the Omani government in collaboration with telecommunication companies made mobile phone calls available for free so people can socialise and support each other. These activities need to be encouraged and adopted by the rest of the countries.

There was an inherited limitation in this study that could not be avoided. The sample composition restricts the potential generalisability of the findings. The distribution of the questionnaires via social media platforms is open to selection bias in terms of recruiting those who are using these tools and are willing to respond to a survey. This limitation was imposed by the restrictions on people's movement and on using paper in the targeted countries. The researchers therefore made a deliberate decision to use the online survey. In the future and when restrictions are removed, it is recommended to replicate the study to include a larger sample and include youth who do not have access to the internet. DASS is not a diagnostic tool for DAS. There is a need for more robust methods to screen youth for mental illness, such as psychiatric interviewing. The cross-sectional design is limited in establishing causal relationship, so there is a need for longitudinal studies addressing these issues more appropriately.

## 5. Conclusion

The current study addressed the current gap in the literature regarding the effects of COVID-19 on the mental health of youth. This was the first study of its kind to collect data from six different countries using a standardised method and one of the few studies to investigate the prevalence of DAS during the COVID-19 pandemic. Healthcare providers, policymakers, and decision-makers should consider the findings and recommendations of this study to establish new emergency plans to address the psychological needs of youths and to prevent and manage emerging mental illness in the future. The three models in the current study predict only 10-11% of variance in DAS among young people. This was expected, considering the complexity of human behaviours and response to stress. There is a need to repeat the current study and include other variables to have a better understanding of depression, anxiety, and stress among youth.

## Figures and Tables

**Table 1:** Sample characteristics (*N* = 1,057).

Variable	*n* (%)	Variable	*n* (%)
Gender		Exposed to person with COVID-19	
Male	301 (28.5)	Yes	14 (1.3)
Female	756 (71.5)	No	1,043 (98.7)
Country		Am I at risk of being infected with COVID-19?	
Oman	155 (14.7)	Yes	437 (41.3)
Jordan	335 (31.7)	No	620 (58.7)
Saudi Arabia	121 (11.4)		
Iraq	117 (11.1)	I have been quarantined for 14 days	
UAE	147 (13.9)	Yes	288 (27.2)
Egypt	182 (17.2)	No	769 (72.8)
Level of education		Do you follow the COVID news?	
9th	29 (2.7)	Yes	980 (92.7)
10th	81 (7.7)	No	77 (7.3)
11th	22 (2.1)	I know someone diagnosed with COVID-19	
12th	163 (15.4)	No	982 (92.9)
University	762 (72.0)	My friend	46 (4.4)
		Family member	29 (3.0)
Do you have a mental illness?		My main source of information about COVID-19 is	
Yes	8 (0.9)	The internet	685 (64.8)
No	1,048 (99.1)	TV	353 (33.4)
Friend diagnosed with mental illness		Friends	19 (1.8)
Yes	54 (5.1)		
No	1,003 (94.9)		
Family member diagnosed with mental illness			
Yes	45 (4.3)		
No	1,011 (95.7)		
Do you have chronic illness			
Yes	91 (8.6)		
No	966 (91.4)		
Variable	*M* (SD)		
Age	21.0 (1.7)		
Depression score	13.2 (10.4)		
Anxiety score	7.6 (7.9)		
Stress score	13.4 (10.4)		
Use of internet before COVID-19 (hours per day)	5.6 (3.8)		
Use of internet after COVID-19 (hours per day)	9.7 (5.0)		

**Table 2:** Prevalence of depression, anxiety, and stress (*N* = 1,057).

Countries	Level	Depression *n* (%)	Anxiety *n* (%)	Stress *n* (%)
Jordan	Normal	147 (44)	204 (60.9)	214 (64)
	Mild	53 (16)	30 (9.0)	31 (9.3)
	Moderate	65 (19.4)	55 (16.6)	36 (10.7)
	Severe	24 (7.3)	11 (3.3)	31 (9.3)
	Extremely severe	46 (13.7)	35 (10.4)	23 (6.9)
Saudi Arabia	Normal	63 (52.1)	81 (66.9)	84 (69.4)
	Mild	14 (11.6)	3 (2.5)	10 (8.3)
	Moderate	16 (13.2)	20 (16.5)	9 (7.4)
	Severe	10 (8.3)	5 (4.1)	10 (8.3)
	Extremely severe	18 (14.9)	12 (9.9)	8 (6.6)
Oman	Normal	68 (43.9)	94 (60.6)	107 (69)
	Mild	26 (16.8)	11 (7.1)	10 (6.5)
	Moderate	28 (18.1)	22 (14.2)	18 (11.6)
	Severe	17 (11)	11 (7.1)	16 (10.3)
	Extremely severe	16 (10.3)	17 (11)	4 (2.6)
Iraq	Normal	49 (41.9)	71 (60.7)	60 (51.3)
	Mild	18 (15.4)	7 (6)	21 (17.9)
	Moderate	24 (20.5)	16 (13.7)	16 (13.7)
	Severe	14 (12)	9 (7.7)	16 (13.7)
	Extremely severe	12 (10.3)	14 (12)	4 (3.4)
UAE	Normal	62 (42.2)	91 (61.9)	85 (57.8)
	Mild	21 (14.3)	10 (6.8)	19 (12.9)
	Moderate	28 (19.0)	19 (12.9)	21 (14.3)
	Severe	19 (12.9)	11 (7.5)	16 (10.9)
	Extremely severe	17 (11.6)	16 (10.9)	6 (4.1)
Egypt	Normal	64 (35.2)	88 (48.4)	104 (57.1)
	Mild	28 (15.4)	13 (7.1)	26 (14.3)
	Moderate	42 (23.1)	40 (22)	22 (12.1)
	Severe	24 (13.2)	16 (8.8)	15 (8.2)
	Extremely severe	24 (13.2)	25 (13.7)	15 (8.2)
Total	Normal	453 (43.0)	629 (59.6)	654 (61.9)
	Mild	160 (15.1)	74 (7)	117 (11.1)
	Moderate	203 (19.2)	172 (16.2)	122 (11.5)
	Severe	108 (10.2)	63 (6.0)	104 (9.8)
	Extremely severe	133 (12.5)	119 (11.2)	133 (12.5)

**Table 3:** Bivariate analysis of depression, anxiety, and stress.

Variable	Category	Depression *M* (SD)	*p*	Anxiety *M* (SD)	*p*	Stress *M* (SD)	*p*
Gender	Male	10.9 (10.0)	<0.001	5.9 (7.2)	<0.001	10.7 (9.7)	<0.001
	Female	14.1 (10.4)		8.3 (8.1)		14.5 (10.4)	
Do you have a family member diagnosed with mental illness	No	12.9 (10.2)	<0.001	7.5 (7.8)	0.056	13.1 (10.1)	<0.001
	Yes	20.1 (12.1)		9.8 (9.6)		19.8 (12.8)	
One friend diagnosed with mental illness	No	12.9 (10.2)	<0.001	7.4 (7.7)	<0.001	13.1 (10.1)	<0.001
	Yes	19.0 (12.3)		11.8 (10.5)		19.3 (12)	
Exposed to person with COVID-19	No	13.1 (10.3)	0.005	7.5 (7.8)	<0.001	13.3 (10.2)	0.002
	Yes	21.0 (13.6)		15 (10.0)		21.9 (10.8)	
I have a relative diagnosed with COVID-19	No	12.9 (10.3)	0.002	7.4 (7.8)	<0.001	13.1 (10.3)	<0.001
	Yes	17.7 (10.5)		12.4 (7.8)		19.1 (9.1)	
I am at risk of being infected with COVID-19	No	11.3 (9.8)	<0.001	6.0 (6.9)	<0.001	11.4 (9.7)	<0.001
	Yes	15.8 (10.5)		9.9 (8.6)		16.2 (10.5)	
I have been quarantined for 14 days	No	12.6 (10.3)	0.002	7.3 (8)	0.027	12.8 (10.3)	0.002
	Yes	14.8 (10.4)		8.5 (7.6)		15.0 (10.2)	

**Table 4:** Linear regression model of independent variables that predict depression, anxiety, and stress.

	Unstandardised coefficient	Standardised coefficient				95% CI for *β*	
	*B*	SE	*β*	*t*	*p*	Lower bound	Upper bound
Model one: predictors of depression							
Constant	-16.160	3.579		-4.515	<0.001	-16.160	3.579
Gender	2.451	0.678	0.106	3.613	<0.001	0.678	2.451
Family member with mental illness	5.357	1.563	0.104	3.427	0.001	5.357	1.563
Friend with mental illness	3.718	1.437	0.079	2.588	0.010	3.718	1.437
Exposed to person with COVID-19	5.883	2.674	0.065	2.200	0.028	5.883	2.674
Risk of having COVID-19	3.786	0.626	0.179	6.049	<0.001	3.786	0.626
Quarantined 14 days	1.456	0.686	0.062	2.122	0.034	1.456	0.686
Using the internet	0.254	0.061	0.122	4.149	<0.001	0.254	0.061
Model two: predictors of anxiety							
Constant	-11.875	2.547		-4.663	<0.001	-16.873	-6.878
Gender	2.002	0.518	0.114	3.868	<0.001	0.987	3.018
Friend with mental illness	3.579	1.060	0.099	3.376	0.001	1.499	5.659
Exposed to person with COVID-19	5.870	2.047	0.085	2.868	0.004	1.853	9.886
Risk of having COVID-19	3.366	0.478	0.209	7.047	<0.001	2.429	4.303
Using the internet	0.162	0.047	0.102	3.466	0.001	0.070	0.254
Model three: predictors of stress							
Constant	-12.188	2.471		-4.933	<0.001	-17.036	-7.339
Gender	3.077	0.668	0.134	4.605	<0.001	1.766	4.389
Family with mental illness	4.849	1.541	0.095	3.147	0.002	1.825	7.872
Friend with mental illness	3.887	1.419	0.083	2.740	0.006	1.103	6.670
Relative with COVID-19	2.154	0.801	0.078	2.688	0.007	0.582	3.727
Risk of having COVID-19	4.327	0.611	0.206	7.077	<0.001	3.128	5.527
Using the internet	0.275	0.061	0.133	4.540	<0.001	0.156	0.394

## Data Availability

The SPSS data used to support the findings of this study are restricted by the Research and Ethics Committee in the College of Nursing at Sultan Qaboos University in order to protect patient privacy. Data are available from Dr. Omar Al Omari, o.alomari@squ.edu.om, for researchers who meet the criteria for access to confidential data.
